# High Prevalence of Antibiotic-Resistant *Escherichia coli* Isolates from Retail Poultry Products in Spain

**DOI:** 10.3390/ani11113197

**Published:** 2021-11-09

**Authors:** Beatriz García-Béjar, Izan García de Blas Martín, María Arévalo-Villena, Ana Briones Pérez

**Affiliations:** 1 Department of Analytical Chemistry and Food Technology, University of Castilla-La Mancha, Camilo José Cela Avenue, 13071 Ciudad Real, Spain; maria.arevalo@uclm.es (M.A.-V.); ana.briones@uclm.es (A.B.P.); 2 Regional Institute of Applied Scientific Investigation (IRICA), University of Castilla-La Mancha, Camilo José Cela Avenue, 13071 Ciudad Real, Spain; izan.garcia1@alu.uclm.es

**Keywords:** antibiotic resistance, enterobacteriaceae, *Escherichia coli*, poultry products, retailers

## Abstract

**Simple Summary:**

Antibiotic resistance happens when bacteria develop the ability to defeat the mechanism of action of chemical compounds designed to kill them. This has become one of the major global concerns in the food chain since it has an effect in diverse steps such as livestock. Poultry products are one of the most consumed type of meat in Spain. In farms, antibiotics are normally used for therapeutic treatments although in the past they were utilized as growth-promoting agents which provoked a high selection pressure in the natural microbiota of fowl. *Escherichia coli* is a gram negative Enterobacteriaceae that is commonly found in chicken microbiota and can be use as interesting indicator of antibiotic resistance in poultry products.

**Abstract:**

The prevalence of *Escherichia coli* was analysed in poultry products from different Spanish retailers and determined its antibiotic resistance capability by phenotypic (ampicillin, amoxicillin, chloramphenicol, gentamicin, imipenem, cefotaxime, tetracycline, ciprofloxacin, trimethoprim, and colistin) and genotypic assays. A total of 30 samples (hindquarters or livers) were collected from supermarkets and butchers. Enterobacteriaceae counts ranged between 3.2 and 6.5 log colony-forming units (CFU)/g, and the highest values were found in livers and in samples from supermarkets. *E. coli* was detected in 83% of the samples tested, and the highest prevalence was observed in livers (100%) and supermarkets (91%). Regarding the antibiotic sensitivity test, 100% of the *E. coli* showed resistance to at least one antibiotic. The highest resistance rates were detected for colistin (87%) and gentamicin (79%), while only two antibiotics (chloramphenicol and cefotaxime) showed a resistance lower than 10%. Furthermore, the resistance genes of tetracycline and beta-lactams were analysed by multiplex PCR, revealing that *tet*(*A*) and *blaTEM* were the majority genes, respectively.

## 1. Introduction

Since 2010, European Union (EU) chicken meat production has grown by 29.8% owing to it being a less-expensive alternative protein and it is perceived as a healthier product. Now, Poland is leading the production market, but the 2nd EU poultry meat producer is Spain (13% in 2021) [1]. Moreover, in the 2019 report issued by the Spanish Ministry of Agriculture (MAPA), it was shown that the consumption of poultry meat has increased more (37.5%) than other types of meat, such as pork (29.8%) and beef (14.6%) [2].

As reported, in the past years the consumer demand for poultry products has increased, which has resulted in continuous pressure on the sector to obtain maximum productivity in minimum time. For this reason, diverse strategies have been developed to increase production, such as genetic selection, innovation in feed products, suitable animal welfare, and biosecurity on farms, as well as a proper supply of antibiotics for the treatment and prevention of infectious diseases [3].

Antibiotics in poultry farms have been employed as therapeutic treatments, prophylactic measures, and growth-promoting agents [3,4,5], among others, although the third use was prohibited on the 1st of January 2006 by the EU owing to the emergence of bacterial resistance [6]. As a consequence of their wrong utilisation, antibiotics have caused a selective pressure for the appearance and spread of antibiotic-resistant bacteria in the chicken biota, allowing the creation of reservoirs of resistance genes [7].

It is important to highlight that diverse zoonotic bacteria are naturally present in the chicken microbiota, such as *Campylobacter* (principally *Campylobacter jejuni* and *Campylobacter coli*), *Salmonella* spp., and *Escherichia coli* [8]. Therefore, when antibiotic resistance occurs in these bacteria, the problem acquires a greater dimension since it can endanger the effect of antibiotics in human medicine [9].

The presence of antibiotic-resistant strains of *E. coli* has been evaluated by diverse authors, which has improved the knowledge of the inadequate use of antibiotics in poultry farms [10]. Both in developing countries and in the main poultry producers, the highest rates of resistance have been detected for beta-lactam antibiotics, tetracyclines, and fluoroquinolones. On the other hand, the lowest rates of resistance have been identified for carbapenems or third-generation cephalosporins. Regarding multi-resistance antibiotics, Spain, Brazil, and China, among others, have a prevalence greater than 80% [11,12,13].

Therefore, the emergence of antibiotic resistance has become a global setback with a great impact on the economy and public health. Therefore, the actions to be taken must be intersectoral and coordinated by the different organisations involved [14].

Accordingly, the main objective of this work was to study the prevalence of phenotype and genotype of antibiotic-resistant *E. coli* isolates from chicken hindquarters and livers acquired from different supermarkets and butchers in Spain.

## 2. Materials and Methods

### 2.1. Sampling

A total of 30 samples of chicken meat were acquired between February and March 2020 from different supermarkets and butchers located in Ciudad Real province (39° N 4° W), a central region of Spain. Of these, 80% were chicken hindquarters, as they are one of the most consumed pieces. The remaining samples (20%) were livers, which have viscera high metabolic importance.

### 2.2. Enterobacteriaceae Counts: Isolation and Identification of Escherichia coli

All the samples were sliced, and 25 g of each sample was homogenised with peptone water (10 g/L) and tween 80 (0.1%) using a masticator (IUL Instruments, Barcelona, Spain). The samples or their decimal dilutions were inoculated in duplicate onto Tryptone Bile X-glucuronide (TBX) agar (Condalab) employing an automatic spiral plater (Eddy Jet 2W, IUL Instruments, Barcelona, Spain). The plates were incubated at 37 °C for 24 h. After that period, colonies were counted, in colony-forming units (CFU), with the automatic colony counter Flash & Go (IUL Instruments, Barcelona, Spain).

Presumptive *E. coli* isolates in TBX agar developed a blue-green colour. Therefore, all these colonies were selected and purified in the same media and subsequently identified by the API 20E test system (Biomeriux) for the elimination of false positives. All *E. coli* isolates were kept at −80 °C with glycerol until they were studied.

### 2.3. Phenotypic Sensitivity Test of E. coli Isolates

With the aim of identifying the sensitivity or resistance of the *E. coli* isolates to diverse antibiotics with clinical interest, a phenotypic test was carried out. A total of ten antibiotic solutions at their minimum inhibitory concentration (MIC) were prepared in Tryptone Soya Broth (TSB) broth (Bioser): ampicillin (32 µg/mL), amoxicillin (32 µg/mL), chloramphenicol (32 µg/mL), gentamicin (16 µg/mL), imipenem (4 µg/mL), cefotaxime (4 µg/mL), tetracycline (16 µg/mL), ciprofloxacin (4 µg/mL), trimethoprim (4 µg/mL), and colistin (2 µg/mL). These doses were selected following the indications given by the Clinical and Laboratory Standards Institute (CLSI, Annapolis, MD, USA).

Cell concentrations from overnight cultures were adjusted to an optical density of 1.2 (OD600). In a 96p microplate, 20 µL of the standardised cultures was inoculated in 180 µL of TSB supplied with each of the antibiotic compounds independently.

The test was carried out in duplicate, and two negative controls were added (TSB broth + *E. coli* isolate without antibiotic and TSB broth + antibiotic without cells). Microplates were incubated at 37 °C/24 h, and absorbance measurements at Time 0 (T0) and Time 24 h (T24) were taken at 600 nm using a plate reader (HiPo MPP-96, Biosan, Riga, Latvia).

#### Growth Curves of Beta-Lactam- and Tetracycline-Resistant *E. coli*

With the obtained results, five *E. coli* isolates were selected randomly among all the isolates previously classified thanks to the phenotypic assay: one sensitive (code 7.1), two with medium resistance (code 6.8 and 7.15), and the other two with high resistance (code 3.10 and 19.27). The assay was carried out as described above, but only the beta-lactam and tetracycline antibiotics were used. In brief, 20 µL adjusted overnight cultures (OD = 1.2) was added to 180 µL of TSB with the selected antibiotics. As negative controls, TSB + antibiotics and TSB + *E. coli* isolates were used and all samples were evaluated by triplicate.

The OD600 of the growth curves was monitored for 24 h at 37 °C using a plate reader (HiPo MPP-96, Biosan, Riga, Latvia) and taking measurements every 30 min which entailed a total of 48 measurements. Before the samples were read, they were agitated for 5 s at 150 rpm. Growth curves were obtained by plotting OD versus time (h). Kinetic parameters were calculated using the model described by Warringer and Blomberg [15]: lag phase (λ), generation time (G), maximum OD (ODmax) and specific growth rate constant (μmax).

### 2.4. Multiplex Polymerase Chain Reaction (PCR) for the Detection of Antibiotic Resistance Genes

The phenotypically confirmed beta-lactam- and tetracycline-resistant *E. coli* were analysed for the presence of resistance genes owing to their importance in the poultry industry.

#### 2.4.1. Beta-Lactam Resistance Genes

A multiplex PCR was carried out to detect the *blaTEM*, *blaSHV*, and *blaCMY-2* genes. For this assay, the primer sequences were selected from Kozak et al. [16] study and were synthesised by Metabion (Germany). The oligonucleotides sequences, their band size and studies in which oligonucleotides were first reported are shown in Table 1. Each 25 µL reaction mix was prepared with a reaction buffer (10×; Biotools), a MgCl2 solution (2.5 mM; Biotools), dNTPs (200 µM; Biotools), a *blaTEM* (0.2 µM) primer, a *blaSHV* (0.4 µM) primer, a *blaCMY-2* (0.2 µM) primer, Taq polymerase (1.25 U/µL; Biotools), and 2.5 µL of extracted DNA. Two controls were included in the reaction, a negative control where the extracted DNA was substituted by Milli-Q water, and a positive control which was *E. coli* with different genes. The amplification process was carried out in a Life Touch thermocycler (Bioer, Hangzhou, China), as follows: 1 initial denaturation cycle at 94 °C/15 min; 30 cycles with the subsequent conditions 94 °C/1 min (denaturation), 55 °C/1 min (hybridisation), and 72 °C/1 min (extension); and a final cycle at 72 °C/10 min (final extension).

PCR products and a 100 bp DNA length standard (Biotools) were loaded in an agarose gel of 1.2% and were subjected to 90 V for 1 h. Fragments were visualised with the gel Green (6×) in a gel documentation system and discrimination was carried out based on the band size.

#### 2.4.2. Tetracycline Resistance Genes

A similar protocol with slight changes was conducted for detecting the *tet*(*A*) and *tet*(*B*) genes (Table 1). Reaction mixes were elaborated as described before, although in this case *tet*(*A*) (0.1 µM) and *tet*(*B*) (0.2 µM) primers were added, as well as 3.5 µL of extracted DNA. The multiplex PCR was performed with the same equipment and under the same conditions as before except that the hybridisation reaction was at 63 °C/1 min. The PCR products were visualised as described in the previous section and the same discrimination process was carried out.

### 2.5. Latex Agglutination Test for E. coli O157:H7

In order to check if any of the antibiotic-resistant *E. coli* isolates were serotype O157:H7, a latex agglutination test was carried out following the manufacture’s indications (Microgen Bioproducts, Camberley, UK).

### 2.6. Statistical Analysis

An analysis of variance (ANOVA) followed by a Duncan test (*p* < 0.05) was performed to determine if significant differences existed in the Enterobacteriaceae mean log CFU/g values of all samples. In that case, no comparaison between groups was carried out—neither anatomical parts nor retailers. The same procedure was carried out with the kinetics parameters. On the other hand, Student’s *t*-test was carried out for comparing Enterobacteriaceae mean log CFU/g values between retailers (supermarkets vs. butchers) and types of samples (hindquarters vs. livers). It was established that significant differences existed when *p* < 0.05.

All the statistical analyses were carried out using the IBM SPSS Statistics program version 24.

## 3. Results

### 3.1. Enterobacteriaceae Counts: Isolation and Identification of Escherichia coli

Counts were ranged between 3.2 and 6.5 log CFU/g (Table 2). The results revealed that the concentration of Enterobacteriaceae was not homogeneous among all chicken samples analysed. This was supported by the ANOVA, which showed that there were significant differences between all the Enterobacteriaceae counts, and by the Duncan test, which grouped the samples into 15 different groups.

Only focusing on the different anatomical parts of the chicken, the liver samples presented counts between 3.6 and 5.4 log CFU/g, while the counts from hindquarters varied between 3.2 and 6.5 log CFU/g. The Student’s *t*-test determined that no significant differences were detected among the counts of the two groups compared (hindquarters and livers).

In the case of retailer’s samples, the Enterobacteriaceae distribution in supermarket samples was heterogenous (3.2–6.5 log CFU/g). A different trend was observed in butchers’ samples, with lower but more homogeneous counts (4.0 and 4.9 log CFU/g). Nevertheless, the Student’s *t*-test indicated that no significant differences existed between supermarkets and butcher’s counts.

All the presumptive *E. coli* isolates were confirmed using the API 20E test system, which corroborated that all isolates belong to this species. A total of 240 *E. coli* isolates were identified in poultry pieces, and they were found in 25 of the 30 samples (Table 2). Samples without *E. coli* were both from supermarkets or butchers, as well as from hindquarters and livers. It was observed that the Enterobacteriaceae counts were not related to the presence or higher proportion of *E. coli*. In fact, the sample with the highest Enterobacteriaceae/*E. coli* proportion (74%) did not present the highest Enterobacteriaceae counts (3.4 Log[CFU/g]). The most usual proportion was ≤10% among all the sample types, except six hindquarters samples and two from livers.

**Figure 1:**
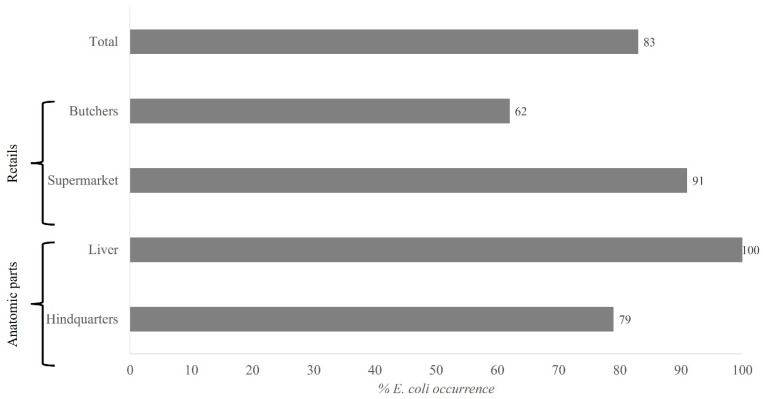
*E. coli* (%) occurrence on the studied samples. All percentages were calculated by dividing the number of samples with *E. coli* presence between the total samples and multiplying by 100.

Regarding the *E. coli* distribution, 83% of the samples were positive, indicating an extensive prevalence in poultry products (Figure 1). It was noticeable that *E. coli* was detected in 91% of the supermarket samples while it was found in only 62% of the butcher samples. On the other hand, all liver pieces and 79% of the hindquarter samples presented *E. coli*.

### 3.2. Phenotypic Sensitivity Test of E. coli Isolates

The optical density difference (∆OD) between the final (ODT24) and initial (ODT0) values allowed the *E. coli* isolates to be classified as sensitive (0.00–0.200), medium resistant (0.201–0.400), or highly resistant (>0.400) to antibiotics [20]. All microorganisms presented some antibiotic resistance, and one was even capable of resisting all 10 antibiotics. Isolates were grouped by their susceptibility or resistance to one or more antibiotics in Table 3 where it can be observed various resistance patterns in the anatomical samples.

The *E. coli* isolates that presented resistance to a smaller number of antibiotics were those sampled from livers bought from supermarkets. It can be also observed that isolates from hindquarters sampled in supermarkets showed resistance to a greater number of antibiotics than those which came from hindquarters sampled in butchers. In fact, for hindquarters sampled in supermarkets, the maximum of *E. coli* isolates documented were able to resist at least six antibiotics while the maximum for those from butchers was detected for three antibiotics. Regarding livers, different resistance trend was documented depending on the retailer of origin. *E. coli* isolates from butchers resisted more antibiotics than isolates from supermarkets.

**Figure 2:**
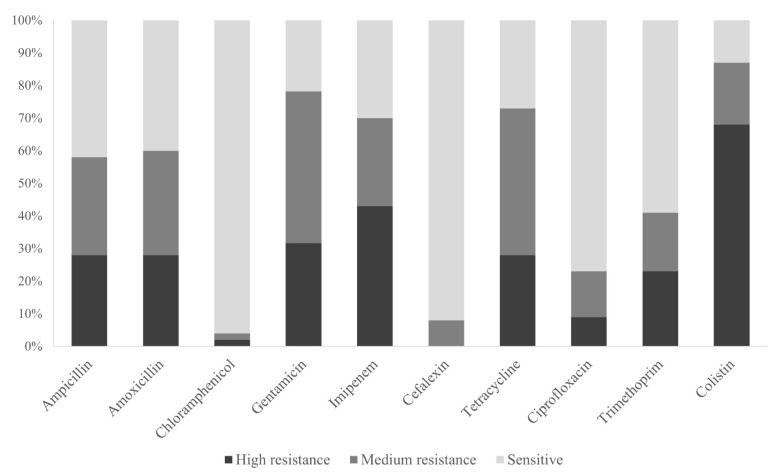
Percentage of sensitive, medium, and high resistance *E. coli* isolates against the tested antibiotics.

The distribution of the *E. coli* isolates (sensitive, medium and high resistance) grown with different antibiotics is shown in Figure 2.

Firstly, the highest resistance value was observed for colistin (87% of the strains showed high resistance). In addition, the resistance rate determined for four antibiotics (colistin, gentamicin, imipenem, and tetracycline) was >60% which are considered high and medium resistance isolates. On the other hand, gentamicin and tetracycline showed more medium-resistant *E. coli* isolates than high-resistant isolates. It was also noticed that the beta-lactam antibiotics tested (ampicillin and amoxicillin) had a similar resistance rate (≈60%) and exhibited a remarkable resistance. In contrast, chloramphenicol and cefotaxime showed the weakest resistance (90% of the strains presented values between 0.00 and 0.200).

**Figure 3:**
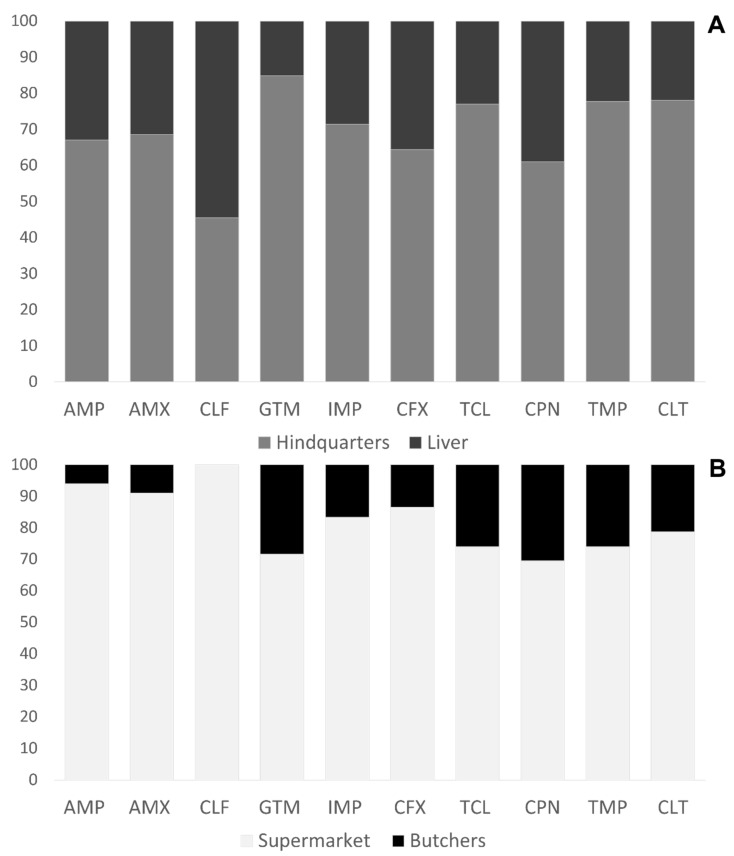
Antibiotic resistant *E. coli* distribution catalogued by poultry products (**A**) and type of retail (**B**).

The distribution of *E. coli* isolates with medium and high antibiotic resistance is shown in Figure 3. In general, the resistance rates were higher in hindquarters than in liver isolates (Figure 3A), except in the case of cefotaxime. Additionally, it was observed that for all antibiotics, the resistance ratio was much higher in supermarket *E. coli* isolates than in other isolates (Figure 3B).

Regarding multi-resistance (resistance against at least three antibiotics), almost 93% of the 240 *E. coli* isolates showed multi-resistance.

#### Growth Curves of Beta-Lactam- and Tetracycline-Resistant *E. coli*

The absorbance measurements were plotted versus time in order to monitor the growth of the *E. coli* isolates against the three antibiotics that are important in poultry farms.

**Figure 4:**
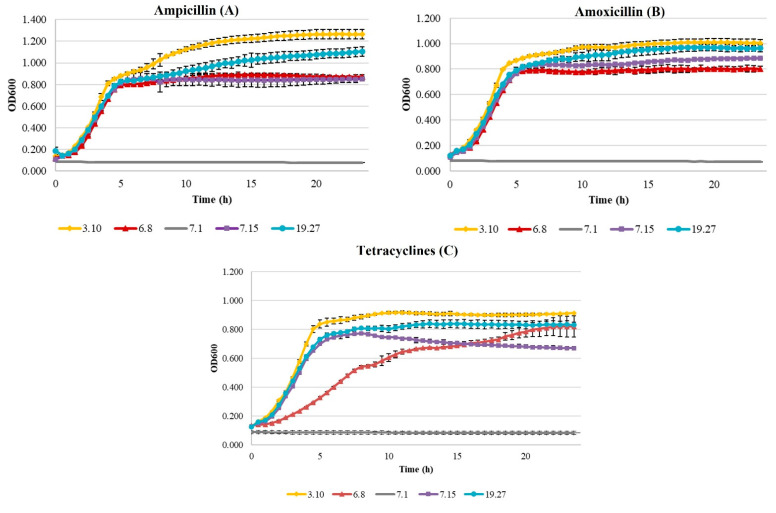
Growth curves of beta-lactam (ampicillin (**A**); amoxicillin (**B**)) and tetracycline (**C**) resistant *E. coli* with high (3.10 and 19.27) and medium resistance (6.8 and 7.15). A sensi isolate was also monetarized (7.1).

As expected, the sensitive (rate between 0.00 and 0.200) *E. coli* isolate (7.1) did not show growth (Figure 4). Regarding beta-lactam, the isolates with the highest resistance (3.10 and 19.27) showed better growth curves than those with medium resistance (6.8 and 7.15) in the presence of ampicillin (Figure 4A). The amoxicillin curves (Figure 4B) showed a similar tendency for the four resistant *E. coli* isolates. In the case of tetracycline (Figure 4C), the best results were observed for the 3.10 isolate, followed by the 19.27 and 7.15 isolates, while the 6.8 isolate presented the slowest growth.

Additionally, four kinetic parameters of all curves were calculated which are collected in Table 4. The ANOVA analysis showed that existed significant differences between the kinetic parameters of the sensitive *E. coli* isolate and those with antibiotic resistance. Regarding medium and high resistance isolates, rate values were ranged between 0.09 h^−1^ (6.8 in tetracycline curves) and 0.19 h^−1^ (3.10 in ampicillin curves) while generation times were between 1.61 h and 3.23 h for the same isolates. These isolates were not separated into groups with significant differences by Duncan’s test, except isolate 3.10 in ampicillin and tetracycline curves. On the other hand, the highest ODmax values and the longest latency phase times were detected both in high resistance isolates (3.10 and 19.27) in each antibiotic tested (Not taking into consideration the sensitive isolate). Moreover, the statistical analysis of these parameters catalogued high and medium resistance isolates in two groups with significant differences between them in the three scenarios.

### 3.3. Multiplex PCR for Detection of Beta-Lactam and Tetracycline Resistance Genes

With the aim of detecting genes related to *E. coli* antibiotic resistance against ampicillin, amoxicillin, and tetracycline, a multiplex PCR with specific primers was carried out. In total, 175 *E. coli* isolates with medium or high tetracycline resistance and 156 isolates with medium or high beta-lactam (ampicillin and amoxicillin) resistance were analysed. The results of the amplification products are shown in Table 5.

Of the three primers tested for beta-lactam antibiotics, only one gene (*blaTEM*) was amplified in all the samples, and multiple amplification or no amplification was not observed. In contrast, 117 of the isolates that were tetracycline resistant (medium and high) presented the *tet*(*A*) gene, while only 16 isolates had the *tet*(*B*) gene, which was the minority gene. Additionally, no amplification was observed in 42 of the samples.

### 3.4. Latex Agglutination Test for E. coli O157:H7

A total of 241 *E. coli* isolates with phenotypic resistance to any of the antibiotics assayed were subjected to this analysis. The latex agglutination test revealed that none of the samples were *E. coli* O157:H7.

## 4. Discussion

An analysis of the prevalence of antibiotic-resistant *E. coli* in poultry products was carried out in this study. For this aim and based on the MAPA information [2], chicken was selected as a raw material since is the main type of meat consumed in Spanish homes (12.4 kg per capita/year). The majority of poultry products consumed in Spain come from lean meat and the hindquarters are the most popular anatomical part. Regarding livers, these foods were selected as representing poultry offal products due to their metabolic importance and ease to be found in stores, although offal consumption is much lower than other product types (0.9 kg per capita/year). Other important factors including the sampling location, supermarkets and butchers were selected because they are the main channels of selling poultry meat. The highest volume of purchases during 2019 in Spain was observed in supermarkets (50.0%) while butchers represented 22.5% of the total. Due to this different consumption importance and distribution, the number of samples from each type of meat and retail was different. More samples were selected from those with greater relevance in the transmission of resistant bacteria (hindquarters and supermarkets). Finally, *E. coli* was chosen among all the diverse Enterobacteriaceae species since is part of the intestinal microbiota of chickens and is considered as an indicator species of antibiotic resistance [21].

The data presented in this study revealed a high prevalence of antibiotic-resistant *E. coli* isolates from poultry products (hindquarters and livers) either from supermarkets or butchers.

The high variability in counts in the poultry pieces could be explained by several factors related to Enterobacteriaceae development, such as the initial cell concentration, intrinsic (i.e., pH, water activity and redox potential) and external (i.e., storage temperature) factors of the food, and the hygienical conditions in the food chain [22]. In fact, it is also usual to observe changes in enteric bacteria concentration in different parts and during various stages of poultry products, which could justify why counts are variable among samples studied [22]. A similar Enterobacteriaceae prevalence was found by Blanco Guarner [23] in different poultry pieces, such as livers, sweetbread, and carcasses. Nevertheless, other authors have reported smaller Enterobacteriaceae populations, although comparable counts were observed in organic chicken meat [24].

Regarding the presence of *E. coli*, similar values were observed by other authors, showing a prevalence of *E. coli* in poultry products between 77% and 100% [12,23,25]. This high prevalence is possible because *E. coli* is part of the chicken microbiota and poultry meat is a nutritive substrate, with a pH and Aw suitable for the development of *E. coli*; moreover, this bacterium can survive long refrigeration times [22]. Therefore, *E. coli* prevalence in poultry products may not be as influenced by geographic origin as other factors such as poultry products that are a favourable matrix for the growth of Enterobacteriaceae.

Strict regulations on the use of antibiotics in meat production have contributed to a drastic decline in the use of these compounds in farms. Fortunately, since the first plan against antimicrobial resistance was established in the EU in 2011, the overall sales of veterinary antibiotics in European countries have decreased by more than 34% [26]. Nevertheless, in the past decades, antibiotics have been used indiscriminately as growth promoters or therapeutical agents, leading to an emergency in the health field [27,28]. This fact was observed in the phenotypic sensitivity test of *E. coli* that indicated the existence of diverse resistance percentages for the antibiotics tested. Our results are similar to those shown in the EFSA and European Centre for Disease Prevention and Control (ECDPC) report on antimicrobial resistance in zoonotic and indicator bacteria from food in 2017 [29].

The resistance percentages of *E. coli* isolates against ampicillin, tetracycline, cefotaxime, ciprofloxacin, trimethoprim, and chloramphenicol were comparable to the values observed in European countries. In the case of cefotaxime and chloramphenicol, which presented the smallest resistance, the same trend has been observed in Belgium (cefotaxime), France, and the United Kingdom (chloramphenicol) [29].

Based on the information collected by the EMA (European Medicines Agency) in the 10th ESVAC (European Surveillance of Veterinary Antimicrobial Consumption) report [16], tetracyclines and penicillins (amoxicillin, ampicillin, and metampicillin) were the most sold group of antibiotics between 2011 and 2018 for animal uses in 31 European countries. This could explain the similar resistance rates observed in diverse EU countries and the present study for these types of antimicrobial compounds [29], except for the amoxicillin results (60%), which were closer to those identified in studies carried out in China and India. Furthermore, the resistance rates of trimethoprim and ciprofloxacin in EU were smaller than those of the previously named antibiotics, probably because fewer doses of trimethoprim and fluoroquinolones were sold for use in slaughtered and livestock animals [26,30].

Progress towards the development of national action plans against antimicrobial resistance appears high in those countries with large livestock sectors. Brazil, Mexico, Argentina, India, Indonesia, Iran, Russia, China, Japan, and the USA are included in the top 10 chicken-producing countries. Among all of them, nine have at least developed a national action plan [31]. However, some of these countries have, for some antibiotics, higher resistance rates in *E. coli* than the rates presented in this study such as India, China and Mexico, owing to their plans to monitor the overuse of antimicrobial compounds are minimal or have been recently established compared to EU countries [32]. Bezerra et al. [13] reported a high prevalence of *E. coli* isolates in broiler chickens, showing resistance to ampicillin (87%), tetracycline (95%), ciprofloxacin (91%), and chloramphenicol (51%). Poultry products from China presented similar resistance values for tetracycline (93%) and chloramphenicol (50%) but higher rates for ampicillin (99%) [11]. Furthermore, other countries, such as Mexico, have reported *E. coli* strains isolated from retail meats with a marked resistance rate against ampicillin (92%), cefotaxime (78%), and tetracycline (75%) [33]. The percentages showed above are higher than those documented in this study, although, as it was commented before, these results were expected since the monitorisation of antibiotics is much lower in these countries and these compounds are extensively used in the food chain in developing countries [32].

Regarding the antibiotics that presented the highest resistance percentage in our study, the *E. coli* isolates showed a notable resistance to gentamicin (80%), which is commonly injected outside the EU in combination with in vivo vaccines to prevent cross-contamination between eggs. It has been documented that this gentamicin-supplemented vaccine could be a critical driver for antimicrobial-resistant bacterial contamination in poultry since it has permitted the adaptation of some bacteria populations to this antibiotic [34,35], so the use of this vaccine may explain the high resistance we found in isolates from livers or hindquarters. In the case of colistin, an increment in resistance has been monitored in recent years in *E. coli* isolates from livestock and slaughtered animals owing to its extensive use which could have resulted in a high selective pressure [36]. Finally, imipenem-resistant bacteria in poultry have been studied in India and Nigeria, where resistance rates of, respectively, 31% and 73% were found in multidrug-resistant beta-lactamase-producing *E. coli*; the latter percentage is in agreement with the data collected in the present study [37,38].

Tetracyclines, together with penicillins in which beta-lactams are included, are currently the most sold drugs for livestock use in Spain [26], so it was interesting to monitor the growth curves of sensitive, medium and high resistance isolates and to carry out a genetic analysis of the principal genes which encoded the resistance to these antibiotics in *E. coli*.

The utilization of growth curves in measuring the effect of antibiotics in bacteria has previously helped in establishing the correlation between bacterial morphology and growth as well as to prove their effect in kinetic parameters [39]. The ODmax and latency phase were the two parameters statistically different between medium and high resistance isolates. This is consistent with other antibiotic tolerance assays where it has been documented that bacteria with longer lag phases are more tolerant to these compounds owing to their prolonged exposition to them at the beginning of the division stage which could lead to a greater final culture density [40]. Therefore, the latency phase seems to be a key kinetic parameter to identify antibiotic resistance isolates as well for knowing their tolerance intensity.

In the case of specific resistance mechanisms developed by bacteria, tetracycline’s resistance mechanism is generally mediated by efflux pumps, which are codified in *E. coli* by *tet* genes, such as *tet*(*A*) and *tet*(*B*) [41]. Similar results were observed by other authors in poultry products, in which the principal gene amplified was *tet*(*A*), while *tet*(*B*) (7%) or both genes (2%) were the least amplified genes [42]. Regarding beta-lactams, they are not efficacious when Gram-negative bacteria are able to produce beta-lactamase enzymes, which can hydrolyse penicillins, among other antibiotics, and which are encoded by the plasmid-mediated *blaTEM*, *blaSHV*, and *blaCTX-M* genes [43]. Other authors have documented that the principal gene detected in beta-lactam-resistant *E. coli* strains was *blaTEM* [11,44]. In fact, Blanco Guarner [23] found similar results to those reported in the present study, and *blaTEM* was amplified in 100% of the ampicillin-resistant *E. coli* strains.

## 5. Conclusions

In conclusion, the Enterobacteriaceae counts were significantly different among the analysed samples. A high prevalence of *E. coli* has been observed both in different poultry products and in poultry meat from different types of retailers. Moreover, the phenotypic test carried out with antibiotics used in the poultry industry has revealed high resistance rates, especially against colistin, gentamicin, imipenem, tetracycline, and beta-lactams.

These circumstances pose a complex global problem that may have a great impact on the economy. Therefore, the emergence of antimicrobial resistance goes beyond the consequences for human and animal health and is becoming a global public health concern, with the need for intersectoral measures coordinated by the different international organisations involved.

## Figures and Tables

**Table 1:** The oligonucleotides sequences, gene band size and study of reference.

Antibiotic Group	Gene	Oligonucleotides	Band Size (pb)	Reference
Beta-lactam	*blaTEM*	5′-TTAACTGGCGAACTACTTAC-3′	247	[16]
		5′-GTCTATTTCGTTCATCCATA-3′		
	*blaSHV*	5′-AGGATTGACTGCCTTTTTG-3′	393	[17]
		5′-ATTTGCTGATTTCGCTCG-3′		
	*blaCMY-2*	5′-GACAGCCTCTTTCTCCACA-3′	1000	[16]
		5′-TGGACACGAAGGCTACGTA-3′		
Tetracycline	*tet*(*A*)	5′-GGCGGTCTTCTTCATCATGC-3′	502	[18]
		5′-CGGCAGGCAGAGCAAGTAGA-3′		
	*tet*(*B*)	5′-CGCCCAGTGCTGTTGTTGTC-3′	173	[19]
		5′-CGCGTTGAGAAGCTGAGGTG-3′		

**Table 2:** Enterobacteriaceae counts (Log[CFU/g]), presence/absence, counts (Log[CFU/g]) and proportion of *E. coli* in the poultry samples studied.

Poultry Product	Retail	Enterobacteriaceae Log[CFU/g]	*E. coli* Presence	*E. coli* Log[CFU/g] (Proportion)
Hindquarters	Supermarket	3.5 ± 0.1 ^c^	+	0.3 ± 0.0 (8%)
		5.2 ± 0.0 ^l^	+	1.5 ± 0.1 (30%)
		5.5 ± 0.0 ^m^	+	0.1 ± 0.0 (2%)
		6.5 ± 0.0 ^ñ^	+	0.2 ± 0.0 (3%)
		3.3 ± 0.1 ^a,b^	+	0.2 ± 0.0 (5%)
		5.2 ± 0.1 ^l^	+	0.8 ± 0.1 (16%)
		6.5 ± 0.0 ^ñ^	-	-
		4.5 ± 0.1 ^h,i^	+	0.1 ± 0.0 (2%)
		4.5 ± 0.0 ^h,i^	+	0.1 ± 0.0 (3%)
		4.4 ± 0.0 ^g,h^	+	0.2 ± 0.0 (5%)
		4.0 ± 0.0 ^e^	+	0.1 ± 0.0 (3%)
		3.4 ± 0.0 ^b,c^	+	2.5 ± 0.1 (74%)
		3.2 ± 0.0 ^a^	+	0.5 ± 0.0 (15%)
		3.5 ± 0.0 ^c^	+	0.1 ± 0.0 (3%)
		4.5 ± 0.0 ^h,i^	+	0.7 ± 0.0 (16%)
		6.2 ± 0.1 ^n^	-	-
		5.0 ± 0.0 ^k^	+	0.1 ± 0.0 (1%)
		4.6 ± 0.1 ^i^	+	0.2 ± 0.0 (5%)
	Butcher	4.3 ± 0.0 ^g^	+	1.4 ± 0.2 (24%)
		4.7 ± 0.1 ^j^	-	-
		4.2 ± 0.1 ^f^	+	0.1 ± 0.0 (2%)
		3.4 ± 0.0 ^b,c^	+	0.1 ± 0.0 (4%)
		3.4 ± 0.0 ^b,c^	+	0.2 ± 0.0 (5%)
		5.0 ± 0.0 ^k^	-	-
Liver	Supermarket	3.7 ± 0.1 ^d^	+	0.2 ± 0.0 (4%)
		5.4 ± 0.0 ^m^	+	0.5 ± 0.1 (9%)
		4.0 ± 0.0 ^e^	+	0.8 ± 0.1 (20%)
		4.5 ± 0.1 ^h,i^	+	0.2 ± 0.0 (5%)
	Butcher	4.0 ± 0.1 ^e^	+	2.3 ± 0.2 (57%)
		5.2 ± 0.1 ^l^	+	0.2 ± 0.0 (3%)

The different superscripts indicate significant differences between simples in the same column (*p* ≤ 0.05).

**Table 3:** Classification of *E. coli* isolates susceptibility or resistance capability among the sampling retailers (supermarket and butcher) and anatomical part (hindquarters and livers) selected.

	Supermarket		Butcher	
Resistance Phenotype	Hindquarters	Livers	Hindquarters	Livers
Suscepetible	0	0	1	0
Resistance to 1	2	0	1	0
Resistance to 2	5	6	3	0
Resistance to 3	18	4	27	0
Resistance to 4	20	5	11	0
Resistance to 5	29	3	6	0
Resistance to 6	38	2	1	4
Resistance to 7	22	1	0	5
Resistance to 8	12	1	0	5
Resistance to 9	6	0	0	2
Resistance to 10	0	0	0	1
Total	152	22	50	17

**Table 4:** Kinetic parameters of the *E. coli* isolates based on their growth curves in presence of ampicillin, amoxicillin or tetracycline (mean values ± standard deviation; *n* = 3).

Antibiotic	*E. coli* Isolate Code	Rate (h^−1^)	Generation Time (h)	ODmax	Latency Phase (h)
Ampicillin	3.10 (high resistance)	0.19 ^c^ ± 0.00	1.61 ^a^ ± 0.01	1.274 ^c^ ± 0.42	1.90 ^b^ ± 0.02
	6.8 (medium resistance)	0.18 ^b^ ± 0.00	1.71 ^b^ ± 0.04	0.891 ^b^ ± 0.01	1.79 ^a^ ± 0.18
	7.1 (sensitive)	0.00 ^a^ ± 0.00	>24 ^c^	0.084 ^a^ ± 0.00	>24 ^c^
	7.15 (medium resistance)	0.17 ^b^ ± 0.00	1.75 ^b^ ± 0.02	0.859 ^b^ ± 0.04	1.77 ^a^ ± 0.00
	19.27 (high resistance)	0.17 ^b^ ± 0.00	1.75 ^b^ ± 0.03	1.099 ^c^ ± 0.05	2.02 ^b^ ± 0.13
Amoxicillin	3.10 (high resistance)	0.18 ^b^ ± 0.01	1.69 ^a^ ± 0.06	1.010 ^c^ ± 0.03	1.94 ^c^ ± 0.01
	6.8 (medium resistance)	0.17 ^b^ ± 0.01	1.75 ^a^ ± 0.13	0.846 ^b^ ± 0.06	1.89 ^b^ ± 0.10
	7.1 (sensitive)	0.00 ^a^ ± 0.00	>24 ^c^	0.082 ^a^ ± 0.00	>24 ^d^
	7.15 (medium resistance)	0.16 ^b^ ± 0.00	1.86 ^b^ ± 0.02	0.904 ^b^ ± 0.13	1.86 ^a,b^ ± 0.04
	19.27 (high resistance)	0.16 ^b^ ± 0.00	1.88 ^b^ ± 0.03	0.972 ^c^ ± 0.03	2.06 ^c^ ± 0.01
Tetracycline	3.10 (high resistance)	0.17 ^d^ ± 0.00	1.78 ^a^ ± 0.01	0.917 ^c^ ± 0.01	2.08 ^b^ ± 0.05
	6.8 (medium resistance)	0.09 ^b^ ± 0.01	3.23 ^c^ ± 0.16	0.800 ^b^ ± 0.07	1.71 ^a^ ± 0.03
	7.1 (sensitive)	0.00 ^a^ ± 0.01	>24 ^d^	0.088 ^a^ ± 0.01	>24 ^c^
	7.15 (medium resistance)	0.15 ^c^ ± 0.07	1.98 ^a,b^ ± 0.04	0.765 ^b^ ± 0.01	1.68 ^a^ ± 0.04
	19.27 (high resistance)	0.14 ^c^ ± 0.01	2.04 ^b^ ± 0.06	0.839 ^c^ ± 0.03	1.96 ^b^ ± 0.13

Different superscripts, in the same column, indicate significant statistical differences (*p* < 0.05) between *E. coli* isolate treated with the same antibiotic, according to Duncan’s test from ANOVA.

**Table 5:** Antibiotic resistance genes detected in *E. coli* isolates with high and medium resistance to beta-lactam and tetracycline.

Antibiotic	Total *E.coli* Resistant Isolates	Amplified Gene	*E. coli*	%
Beta-lactam (ampicillin and amoxicillin)	156	*bla* *SHV*	0	0
		*bla* *TEM*	156	100
		*bla* *CMY-2*	0	0
		All genes	0	0
		No gene	0	0
Tetracycline	176	*tet*(*A*)	117	67
		*tet*(*B*)	16	9
		All genes	0	0
		No gene	42	24

## Data Availability

Not applicable.

## References

[1] European Commission (2021). Poultry: Information on an Overview of EU Poultry, Market Measures and Standards, Trade Measures, Market Monitoring, Legal Bases and Committees. https://ec.europa.eu/info/sites/default/files/food-farming-fisheries/farming/documents/poultry-meat-dashboard_en.pdf.

[2] MAPA (2020). Informe del Consumo Alimentario en España 2019. https://www.mapa.gob.es/en/alimentacion/temas/consumo-tendencias/informe2019_v2_tcm38-540250.pdf.

[3] Muaz K., Riaz M., Akhtar S., Park S., Ismail A. (2018). Antibiotic residues in chicken meat: Global prevalence, threats, and decontamination strategies: A review. J. Food Protect..

[4] Mund M.D., Khan U.H., Tahir U., Mustafa B.E., Fayyaz A. (2017). Antimicrobial drug residues in poultry products and implications on public health: A review. Int. J. Food Propert..

[5] Manyi-Loh C., Mamphweli S., Meyer E., Okoh A. (2018). Antibiotic use in agriculture and its consequential resistance in environmental sources: Potential public health implications. Molecules.

[6] Anadón A. (2006). The EU ban of antibiotics as feed additives (2006): Alternatives and consumer safety. J. Vet. Pharm. Therap..

[7] Witte W. (2000). Selective pressure by antibiotic use in livestock. Int. J. Antimicrob. Agents.

[8] Schroeder C.M., White D.G., Meng J. (2004). Retail meat and poultry as a reservoir of antimicrobial-resistant *Escherichia coli*. Food Microbiol..

[9] Clavijo V., Flórez M.J.V. (2018). The gastrointestinal microbiome and its association with the control of pathogens in broiler chicken production: A review. Poult. Sci..

[10] EFSA (2017). The European Union Summary Report on Antimicrobial Resistance in Zoonotic and Indicator Bacteria from Humans, Animals and Food in 2017. https://efsa.onlinelibrary.wiley.com/doi/epdf/10.2903/j.efsa.2019.5598.

[11] Jiang H.X., Lü D.H., Chen Z.L., Wang X.M., Chen J.R., Liu Y.-H., Liao X.P., Liu J.H., Zeng Z.L. (2011). High prevalence and widespread distribution of multi-resistant *Escherichia coli* isolates in pigs and poultry in China. Vet. J..

[12] Ghodousi A., Bonura C., Di Noto A.M., Mammina C. (2015). Extended-spectrum ß-Lactamase, AmpC-Producing, and fluoroquinolone-resistant *Escherichia coli* in retail broiler chicken meat, Italy. Foodborne Pathog. Dis..

[13] Bezerra W.G.A., da Silva I.N.G., Vasconcelos R.H., Machado D.N., Lopes E.D.S., Lima S.V.G., de Teixeira R.S.C., Lima J.B., Oliveira F.R., Maciel W.C. (2018). Isolation and antimicrobial resistance of *Escherichia coli* and *Salmonella enterica* subsp. Enterica (O: 6, 8) in broiler chickens. Acta Sci. Vet..

[14] PRAN (2019). Plan Nacional Frente a la Resistencia a los Antibióticos 2019–2021. http://www.resistenciaantibioticos.es/es/system/files/field/files/pran_2019-2021_0.pdf?file=1&type=node&id=497&force=0.

[15] Warringer J., Blomberg A. (2003). Automated screening in environmental arrays allows analysis of quantitative phenotypic profiles in *Saccharomyces cerevisiae*. Yeast.

[16] Kozak G.K., Boerlin P., Janecko N., Reid-Smith R.J., Jardine C. (2009). Antimicrobial resistance *in Escherichia coli* isolates from Swine and wild small mammals in the proximity of swine farms and in natural environments in Ontario, Canada. Appl. Environ. Microbiol..

[17] Travis R.M., Gyles C.L., Reid-Smith R., Poppe C., McEwen S.A., Friendship R., Janecko N., Boerlin P. (2006). Chloramphenicol and kana-mycin resistance among porcine *Escherichia coli* in Ontario. J. Antimicrob. Chemother..

[18] Lanz R., Kuhnert P., Boerlin P. (2003). Antimicrobial resistance andresistance gene determinants in clinical *Escherichia coli* from different ani-mal species in Switzerland. Vet. Microbiol..

[19] Goswami P.S., Gyles C.L., Friendship R.M., Poppe C., Kozak G.K., Boerlin P. (2008). Effect of plasmid pTENT2 on severity of porcine post-weaning diarrhoea induced by an O149 enterotoxigenic *Escherichia coli*. Vet. Microbiol..

[20] Fernández-Pacheco P., García-Béjar B., Jiménez-del Castillo M., Carreño-Domínguez J., Briones Pérez A., Arévalo-Villena M. (2020). Potential probiotic and food protection role of wild yeasts isolated from pistachio fruits (*Pistacia vera*). J. Sci. Food Agric..

[21] Roth N., Käsbohrer A., Mayrhofer S., Zitz U., Hofacre C., Domig K.J. (2019). The application of antibiotics in broiler production and the resulting antibiotic resistance in *Escherichia coli*: A global overview. Poultry Sci..

[22] Jay J.M., Loessner M.J., Golden D.A. (2005). Modern Food Microbiology.

[23] Blanco Guarner N. (2019). Detección de Cepas Multirresistentes de Escherichia coli Mediante Análisis de Resistencia Fenotípica y Genotípica, en Productos Avícolas Destinados a Consumo Humano. https://riunet.upv.es/handle/10251/126824.

[24] Miranda J., Vazquez B., Fente C., Barros-Velázquez J., Cepeda A., Franco C. (2008). Antimicrobial resistance in *Escherichia coli* strains isolated from organic and conventional pork meat: A comparative survey. Eur. Food Res. Technol..

[25] Hussain A., Shaik S., Ranjan A., Nandanwar N., Tiwari S.K., Majid M., Baddam R., Qureshi I.A., Semmler T., Wieler L.H. (2017). Risk of transmission of antimicrobial resistant *Escherichia coli* from commercial broiler and free-range retail chicken in India. Front. Microbiol..

[26] EMA, ESVAC (2020). Sales of Veterinary Antimicrobial Agents in 31 European Countries in 2018. https://www.ema.europa.eu/en/documents/report/sales-veterinary-antimicrobial-agents-31-european-countries-2018-trends-2010-2018-tenth-esvac-report_en.pdf.

[27] EMA, ESVAC (2018). Sales of Veterinary Antimicrobial Agents in 30 European Countries in 2016. https://www.ema.europa.eu/en/documents/report/sales-veterinary-antimicrobial-agents-30-european-countries-2016-trends-2010-2016-eighth-esvac_en.pdf.

[28] Hao Van T.T., Yidana Z., Smooker P., Coloe P. (2019). Antibiotic use in food animals in the world with focus on Africa: Pluses and minuses. J. Glob Antimicrob. Resist..

[29] EFSA, ECDC (2020). The European Union summary report on antimicrobial resistance in zoonotic and indicator bacteria from humans, animals and food in 2017/2018. EFSA J..

[30] Abbassi M.S., Kilani H., Zouari M., Mansouri R., Oussama E.F., Hammami S., Chehida N.B. (2017). Antimicrobial resistance in *Escherichia coli* isolates from healthy poultry, bovine and ovine in Tunisia: A real animal and human health threat. J. Clin. Microbiol. Biochem. Technol..

[31] WHO, FAO, OIE (2018). Monitoring Global Progress on Addressing Antimicrobial Resistance: Analysis Report of the Second Round of Results of AMR Country Self-Assessment Survey 2018. World Health Organization. https://apps.who.int/iris/handle/10665/273128.

[32] Founou L.L., Founou R.C., Essack S.Y. (2016). Antibiotic Resistance in the Food Chain: A Developing Country-Perspective. Front Microbiol..

[33] Martínez-Vázquez A.V., Rivera-Sánchez G., Lira-Méndez K., Reyes-López M.A., Bocanegra-García V. (2018). Prevalence, antimicrobial resistance and virulence genes of *Escherichia coli* isolated from retail meat in Tamaulipas, Mexico. J. Glob. Antimicrob. Res..

[34] Dutil L., Irwin R.J., Finley R., Ng L.K., Avery B.P., Boerlin P., Bourgault A.M., Cole L., Daignault D., Desruisseau A. (2010). Ceftiofur resistance in *Salmonella enterica* serovar Heidelberg from chicken meat and humans, Canada. Emerg. Infect. Dis..

[35] Davis G.S., Waits K., Nordstrom L., Grande H., Weaver B., Papp K., Horwinski J., Koch B., Hungate B.A., Liu C.M. (2018). Antibiotic-resistant *Escherichia coli* from retail poultry meat with different antibiotic use claims. BMC Microbiol..

[36] Liu Y.Y., Wang Y., Walsh T.R., Yi L.X., Zhang R., Spencer J., Doi Y., Tian G., Dong B., Huang X. (2016). Emergence of plasmid-mediated colistin resistance mechanism MCR-1 in animals and human beings in China: A microbiological and molecular biological study. Lancet Infect. Dis..

[37] Kar D., Bandyopadhyay S., Bhattacharyya D., Samanta I., Mahanti A., Nanda P.K., Mondal B., Dandapat P., Das A.K., Dutta T.K. (2015). Molecular and phylogenetic characterization of multidrug resistant extended spectrum beta-lactamase producing *Escherichia coli* isolated from poultry and cattle in Odisha, India. Infect. Genet. Evol..

[38] Chika E., Ifeanyi I., Amaechi C., Malachy U., Peter E., Chidinma I., Ogene L., Orinya C. (2017). Multiple antibiotic resistance, antibiogram and phenotypic detection of Metallo-Beta-Lactamase (MBL) from *Escherichia coli* of poultry origin. J. Appl. Microbioliol. Biochem..

[39] Yourassowsky E., Van der Linden M.P., Lismont M.J., Crokaert F., Glupczynski Y. (1985). Correlation between growth curve and killing curve of *Escherichia coli* after a brief exposure to suprainhibitory concentrations of ampicillin and piperacillin. Antimicrob. Agents Chemother..

[40] Bertrand R.L. (2019). Lag Phase Is a Dynamic, Organized, Adaptive, and Evolvable Period That Prepares Bacteria for Cell Division. J. Bacteriol..

[41] Li X.Z., Nikaido H. (2009). Efflux-mediated drug resistance in bacteria: An update. Drugs.

[42] Vuthy Y., Lay K.S., Seiha H., Kerleguer A., Aidara-Kane A. (2017). Antibiotic susceptibility and molecular characterization of resistance genes among *Escherichia coli* and among *Salmonella* subsp. in chicken food chains. Asian Pac. J. Trop. Biomed..

[43] Maamar E., Hammami S., Alonso C.A., Dakhli N., Abbassi M.S., Ferjani S., Hamzaoui Z., Saidani M., Torres C., Boutiba-Ben Boubaker I. (2016). High prevalence of extended-spectrum and plasmidic AmpC beta-lactamase-producing *Escherichia coli* from poultry in Tunisia. Int. J. Food Microbiol..

[44] Ugbo E.N., Anyamene C.O., Moses I.B., Iroha I.R., Babalola O.O., Ukpai E.G., Chukwunwejim C.R., Egbule C.U., Emioye A.A., Okata-Nwali O.D. (2020). Prevalence of blaTEM, blaSHV, and blaCTX-M genes among extended spectrum beta-lactamase-producing *Escherichia coli* and *Klebsiella pneumoniae* of clinical origin. Gene Rep..

